# Coronavirus disease and male fertility: a systematic review

**DOI:** 10.1186/s43043-021-00073-4

**Published:** 2021-08-16

**Authors:** Mobina Fathi, Kimia Vakili, Abbas Aliaghaei, Shahrzad Nematollahi, Tahmineh Peirouvi, Ali Shalizar-Jalali

**Affiliations:** 1 Student Research Committee, Faculty of Medicine, Shahid Beheshti University of Medical Sciences, Tehran, Iran; 2 Faculty of Medicine, Shahid Beheshti University of Medical Sciences, Tehran, Iran; 3 Men’s Health and Reproductive Health Research Center, Shahid Beheshti University of Medical Sciences, Tehran, Iran; 4 Department of Histology and Embryology, Faculty of Medicine, Urmia University of Medical Sciences, Urmia, Iran; 5 Department of Basic Sciences, Faculty of Veterinary Medicine, Urmia University, Urmia, Iran

**Keywords:** Coronavirus disease, Angiotensin-converting enzyme, Testis, Sperm, Male fertility

## Abstract

**Background:**

Based on the information from other SARS-CoV infections in the patients recovered from COVID-19, particularly cases in the reproductive age, gonadal function evaluation and andrological consultation comprising semen analysis are recommended.

**Main body:**

Based on the COVID-19 infected patients’ seminal fluid analyses, SARS-CoV-2 may employ the male reproductive system as a transmission pathway. It has been also demonstrated that angiotensin-converting enzyme 2 (ACE2) can be strongly expressed at the protein levels in the testicular cells. The high expression of ACE2 in testes suggests that testes in the COVID-19 infected males can have an important role in the viral persistence and this subject needs further investigations. Several researchers have examined males recovered from COVID-19, but still, large-scale experiments are needed to determine the effects of SARS-CoV-2 on the male reproductive system as well as viral transmission risk.

**Conclusion:**

Comprehensive researches are required to figure out the presence of the SARS-CoV-2 virus in seminal fluid as well as its sexual transmissibility and impact on sperm characteristics.

## Background

The World Health Organization has announced coronavirus disease 2019 (COVID-19) as a global pandemic since March 11, 2020 [1]. The pandemic, also known as the SARS-CoV-2 pandemic, has imposed huge pressure on health care systems around the world along with negative social and economic consequences [2]. Although the precise effects of SARS-COV-2 on the urinary tract have not yet been identified, this does not mean that urinary specialists will not investigate about this subject [3].

In order to restrict the spread of the COVID-19 pandemic, investigations on the transmission routes and mechanisms are extensively conducted. The SARS-CoV-2 mainly transmits through respiratory droplets [4] and has been also observed in different biological fluids including blood, urine, and feces [5]. The presence of SARS-CoV-2 in genital secretions has not been confirmed yet. Due to the blood-testis barrier (BTB), testis is partially immune to many microorganisms [6]; however, some viruses such as the mumps virus have the ability to cross the BTB and cause localized testis inflammation in forms of orchitis [7]. On the other hand, angiotensin-converting enzyme 2 (ACE2) and viral spike (S) protein, the mediators for SARS–COV-2 entrance into the target cells, mainly exist in human testis [8, 9]. These observations have raised concerns over the possibility of sexual transmission of COVID-19 [10], although the evidence is largely insufficient [11].

Confirmation about the existence of SARS-CoV-2 in the seminal fluid would bear new and serious reproductive and sexual insights into this pandemic [12, 13].

The present study aimed to review the evidence regarding presence of SARS–CoV-2 in seminal fluid and to define the expression profile of trans-membrane protease, serine 2 (TMPRSS2), and ACE2 in human testes to provide beneficial insights into the viral entry and the early impacts of the virus on male reproductive functions. This systematic review will shed light on the possibility of SARS-CoV-2 transmission through semen and reproductive complications following infection, which might influence fertility in young COVID-19 patients.

## Main text

**Fig. 1.**
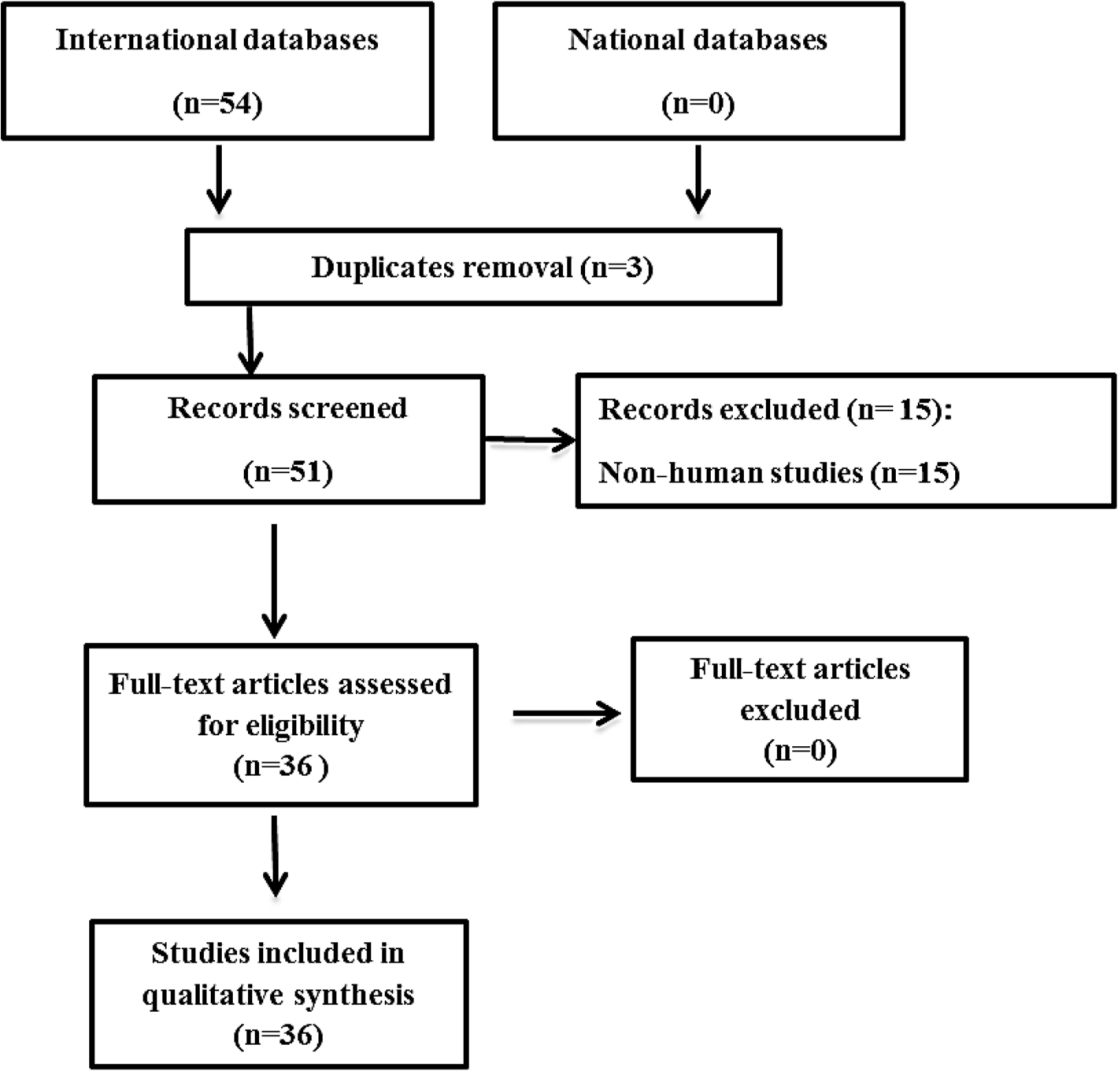
Diagram of included studies into the systematic review

The present study aimed to systematically review the current literature regarding the presence of SARS-CoV-2 in seminal fluids. International scientific databases including Scopus, Web of Science, and MEDLINE as well as national databases including Meh Iran, SID, and Iran Doc were reviewed with the following keywords: SARS-CoV-2, COVID-19, coronavirus, ACE, angiotensin II, angiotensin-converting enzyme 2, Ang-(1-7), testes, male reproductive system, male fertility, and semen. Inclusion criteria were defined as all published papers in Persian or English languages that studied the presence of coronavirus in seminal fluids of the male human host. Exclusion criterion was defined for articles that assessed types of coronaviruses other than SARS-CoV-2. Selection of papers was performed by one researcher, while the second researcher reviewed the records to exclude duplicates. After reviewing titles of 51 studies, 15 were removed due to non-human subjects. Finally, data of 36 papers including author information, study location, virus detection method, and study findings were extracted by two independent researchers (Fig. 1). Due to the huge variability in the study findings and methods, we could not perform meta-analysis in the included studies.

### Angiotensin-converting enzyme 2 expression in testes

The SARS-CoV2 enters host cells through a complex process, starting with the interaction of its spike protein with ACE2 [8, 14]. The ACE2 is a monocarboxypeptidase, converting angiotensin II to angiotensin 1-7. These factors (angiotensin 1-7) can stimulate MAS receptors and antagonize the effects of angiotensin I [15].

The SARS-CoV-2 shares 80% sequence homology and cell adhesion via ACE2 with another coronavirus, SARS-CoV being responsible for SARS outbreak in 2003 [16]. However, SARS-CoV-2 has some distinct characteristics increasing its clinical virulence. For instance, a more compact configuration in the three-dimensional structure of this virus enhances its binding affinity to ACE2 as a viral receptor. Additionally, SARS-CoV-2 has a polybasic cleavage site increasing its viral internalization into the cells. According to these unique features of SARS-CoV-2, the stronger virulence of this virus compared to previous coronaviruses is partially justifiable [17].

The ACE2 has a high expression level in various human tissues and organs including lung alveolar epithelial cells, small intestinal epithelial cells, vascular endothelial cells, smooth muscle cells, brush border of proximal tubular cells, and parietal epithelial cells in kidneys and oral and nasal mucosae [18]. Moreover, ACE2 is highly expressed in human testes. Specifically, ACE2 is expressed in seminiferous tubules cells along with Leydig and Sertoli cells in testes, which might be associated with potential COV infection in the reproductive system [19]. Interestingly, ACE2 expression in testes is age-dependent. Accordingly, the highest and lowest expressions of ACE2 occur in patients aged 30 and 60, respectively [20]. Another probable cause for localization of SARS-CoV-2 in the male reproductive system can be the expression by furin domains in human’s epididymis, as described above [21].

It has been proved that cells expressing TMPRSS2 for S protein (which can initiate the viral internalization process) on their surface have enhanced SARS-CoV-2 isolation [14]. The TMPRSS2 is highly expressed in the epithelial cells of the human prostate gland, especially, in the apical surface of the luminal cells. The expression of TMPRSS2 is controlled by androgens (the promoter enhancers of the *TMPRSS2* gene). The TMPRSS2 can be released into semen via proteasomes, which are vesicles that may help sperms to have better function [22]. The high TMPRSS2 expression in the prostate can be related to COV infection and another point of weakness of the male reproductive system.

### The presence of the SARS-CoV-2 virus in the semen and testis

If SARS-CoV-2 employs SARS-CoV receptor ACE2 to enter the cell, TMPRSS2 is repairing this process and thus, the advanced separation of SARS-CoV-2 in the cells expressing TMPRSS2 may occur [13]. High expression of TMPRSS2 is observed in prostate epithelial cells and includes the apical plasma membrane of luminal cells located in the prostate. Its expression can be regulated by androgens, which seems to be enhancers of the gene and additionally could be released into the semen as a component of proteasomes. It also has the ability to improve male reproduction ability and to enhance the sperm’s function [22].

The presence of SARS-CoV-2 in sperm and seminal fluid and the likelihood of COVID-19 transmission through sexual intercourse need to be investigated. Despite observed considerable damage to seminiferous tubules and Sertoli cells, mild inflammation in the interstitium, and reduction in the Leydig cell population [23], attempts to find signs of SARS-CoV-2 in seminal fluid have resulted in contradictory findings [24].

The SARS-CoV-2 may influence the production of luteinizing hormone, follicle-stimulating hormone, and testosterone as well as the testes [25]. In one small-scale study, SARS-CoV-2 was detected in semen in 26.70% of COVID-19 patients in the acute phase and 8.70% of the patients in the recovery phase [26]. On the other hand, failure to detect SARS-CoV-2 in the seminal fluid of patients in clinical or recovery phases of COVID-19 is frequently reported [21, 27]. Search for SARS-CoV-2 in the seminal fluid of asymptomatic or deceased COVID-19 patients also did not lead to any promising findings [23, 28]. The different findings among studies might partially be attributed to various phases of disease course, clinical condition of patients, and detection method such as RT-PCR and electron microscopy.

### SARS-CoV-2 and testis: similarity with SARS

As stated before, SARS-CoV-2 shares a lot of mutual characteristics with SARS-CoV. In 2003, orchitis (inflammation of the testicles) was considered as a complication in SARS patients [29] following a study on six cases with SARS-associated fatal orchitis. Several mechanisms have been proposed to explain the underlying cause of testicular damage in SARS patients, which can logically be applicable to the SARS-CoV-2 as well. These hypotheses are as follow:

1. The ideal temperature for spermatogenesis is 1–8 °C lower than regular human body temperature (37 °C). Long-term fever can lead to increased testicular temperature and cause germ cells destruction and degeneration. According to previous studies, high temperature can also result in germ cells apoptosis [30]. High fever is frequently reported in both SARS and COVID-19 patients. Therefore, it might have a direct effect on testicular destruction.
2. Steroids are one of the medication options for SARS and COVID-19 patients, despite their clinical efficacy is still debating [31]. However, previous evidence did not support this hypothesis, as one of the cases who had not received glucocorticoid developed orichitis in the same way as others [29].
3. Viruses may also be able to directly infect testis. Considering the earlier discussion about the role of ACE2 in viral entrance of SARS-CoV and SARS-CoV-2, infection of testis by viruses seems plausible. However, studies have represented conflicting results about possibility of direct SARS virus infection in testis. According to Xu et al., none of six cases represented signs of direct infection in their testes [29]. While in the study conducted by Zhao et al., SARS-CoV was present in Leydig and testicular germ cells [32]. As SARS-CoV-2 enters host cells via ACE2, the risk of direct testicular infection exists in COVID-19 patients; however, the evidence is not found yet.
4. Leukocyte infiltration in testis can interfere with the Leydig cells’ normal function and testosterone production and then lead to BTB damage and seminiferous epithelium destruction. Leukocytes can also lead to autoantibody development and autoimmune responses in seminiferous tubules via inflammatory cytokines releasing [29]. Increase of serum IgG levels is reported in both SARS and COVID-19 patients [33]. Therefore, SARS-CoV and SARS-CoV-2 may be able to trigger secondary autoimmune responses in testis and cause autoimmune orchitis.
5. Viral infections result in redox homeostasis disruption in the body leading to reactive oxygen species (ROS) over-production building an ideal situation for viral replication [34]. In line with that, coronavirus infections such as SARS-CoV and SARS-CoV-2 can cause male reproductive dysfunction through ROS over-generation and apoptotic pathways activation [35, 36].

## Conclusions

**Fig. 2.**
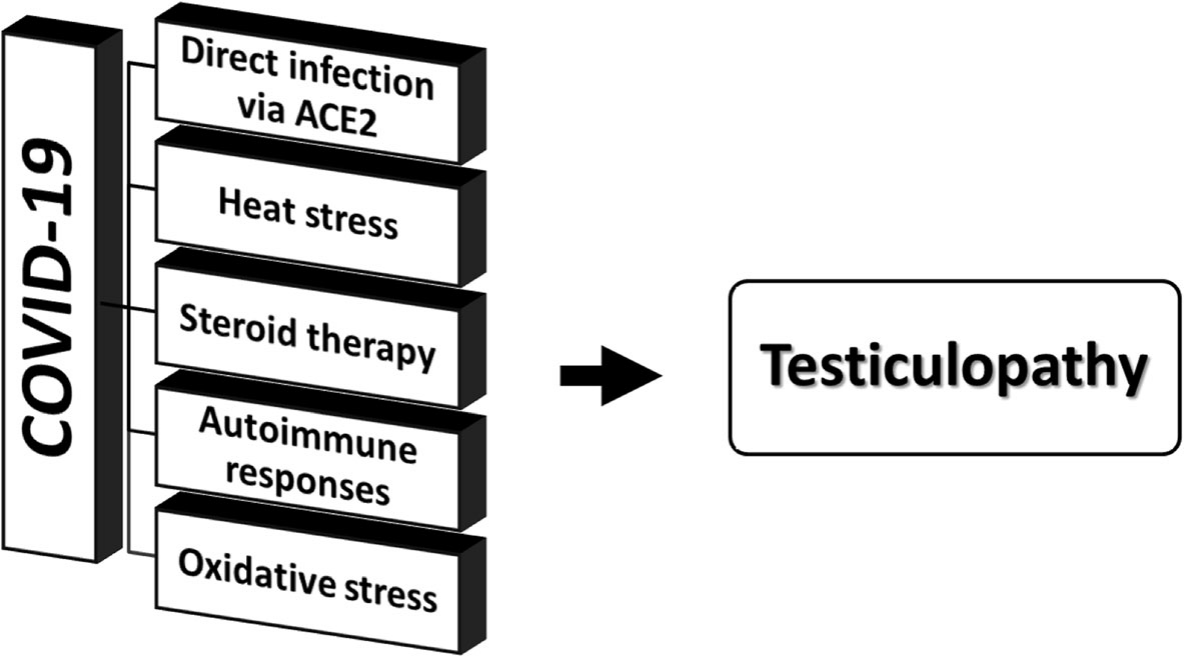
Possible mechanisms of COVID-19-induced testicular damage

Testicular damage has been reported following SARS-CoV-2 infection, while clinical damage of the virus is theoretically possible. The hypothetical mechanisms for this damage include direct viral invasion to testicular tissue through ACE2 receptors, temperature-related testicular damage as a result of persistent high fever, secondary inflammatory and autoimmune responses, unexpected side effects of COVID-19 medications such as steroids, and viral infection-related oxidative stress (Fig. 2). On the other hand, clinical and epidemiological evidence regarding the effects of COVID-19 on reproductive health and future infertility of male patients is hugely scarce. As COVID-19 is a newly emerged disease, further follow-up studies on reproductive outcomes of recovered patients (especially those who are of reproductive age) are recommended to investigate probable long-term consequences.

## Data Availability

Not applicable

## Abbreviations

ACE2: Angiotensin-converting enzyme 2
COVID-19: Coronavirus disease 2019
BTB: Blood-testis barrier
TMPRSS2: Transmembrane protease, serine 2
ROS: Reactive oxygen species
